# Illness of Dr. M. H. Webb

**Published:** 1882-08

**Authors:** 


*Illness of Dr. Marshall H. Webb.*—The many friends of Dr. Marshall H. Webb, will be pained to know that he has been lying seriously ill for a long time at his home in Lancaster, Pa., and that his condition at the time of this writing is deemed critical. It is earnestly hoped that these apprehensions may prove groundless, and that the "little Doctor" may be soon with us again, in health.

---

